# *BRAF*, *KRAS* and *PIK3CA* Mutation and Sensitivity to Trastuzumab in Breast Cancer Cell Line Model 

**DOI:** 10.31557/APJCP.2020.21.1.1

**Published:** 2020

**Authors:** Ebubekir Dirican

**Affiliations:** 1 *Istanbul Aydın University, Vocational School of Health Services,*; 2 *Marmara University, School of Medicine Department of Medical Biology, Istanbul, Turkey. *

**Dear Editor**

I have read with interest the article in this journal by Patra et al., (2017) concerning* BRAF*, *KRAS* and *PIK3CA* mutation and sensitivity to trastuzumab in breast cancer cell line model. They evaluated trastuzumab responsiveness in breast cancer (BCa) cell lines. They showed the optimum concentration of trastuzumab to be 7 μg/well. Moreover, they detected BRAF and KRAS mutated cell line, MDA-MB-231, found the least sensitivity after being treated with trastuzumab when to the sensitivity of the *PIK3CA* mutated cell lines, *MCF-7 *and *MDA-MB-361*, and *KRAS-BRAF-PIK3CA* cell line, *MDA-MB-453*.

Phosphatidylinositol-4,5-bisphosphate 3 kinase catalytic subunit α (*PIK3CA*), which is located on chromosome 3, encodes the catalytic subunit p110α of class IA phosphoinositide 3-kinase (*PI3K*). Also, *PIK3CA* is one of the most commonly mutated oncogenes in several types of human cancer, including breast, colon and endometrial cancer (Cizkova et al., 2013; Dirican et al., 2016). In 2014, we aimed *PIK3CA* mutations in BCa investigating clinical and prognostic significances (Dirican et al., 2014). And frequency of *PIK3CA* mutations found 31%, should be diagnostic significance (Dirican et al., 2014). We believe the potential of *PIK3CA* mutations as an important biomarker for BCa classification and the possible use of *PIK3CA* inhibitor as targeted therapy for BCa. Therefore, we need to increase the number of trials on the *PIK3CA* gene in vivo and in vitro. In the future, these trials will help to choice correct treatment models. Patra et al., (2017) showed that the cell line most sensitivite to trastuzumab is *MDA-MB-453 *with the *PIK3CA*. This findings are important which indicates the potential mechanism for trastuzumab resistance. Especially, HER-2 positive BCa cells were treated with combining trastuzumab with a *PIK3CA* inhibitor agent. A recent study reported that *PIK3CA (E542K)* were found to be significantly associated with reduced disease-free survival (DFS) in patients treated with trastuzumab (p: 0.018 and 0.005, respectively) (Singla et al., 2019). This study implied that *PIK3CA* genes are important biomarkers in HER2+ BCa. Also, the patients harboring mutant *PIK3CA* exhibit a poorer clinical outcome as compared to those carrying wild-type *PIK3CA*. *PIK3CA* mutation analysis can be usefully performed in real-life clinical practice (Cociolone et al., 2018). Investigation of the molecular mechanism of *PIK3CA* and its related genes is important for BCa treatment.

## References

[B1] Cocciolone V, Cannita K, Tessitore A (2018). Neoadjuvant chemotherapy in breast cancer: a dose-dense schedule in real life and putative role of PIK3CA mutations. Oncotarget.

[B2] Cizkova M, Dujaric ME, Lehmann-Che J 2013) Outcome impact of PIK3CA mutations in HER2-positive breast cancer patients treated with trastuzumab. Br J Cancer.

[B3] Dirican E, Akkiprik M, Ozer A (2016). Mutation distributions and clinical correlations of PIK3CA gene mutations in breast cancer. Tumour Biol.

[B4] Dirican E, Kaya Z, Gullu G (2014). Detection of PIK3CA gene mutations with HRM analysis and association with IGFBP-5 expression levels in breast cancer. Asian Pac J Cancer Prev.

[B5] Patra S, Young V, Llewellyn L, Senapati JN, Mathew J (2017). BRAF, KRAS and PIK3CA mutation and sensitivity to Trastuzumab in breast cancer cell line model. Asian Pac J Cancer Prev.

[B6] Singla H, Kaur RP, Shafi G (2019). Genomic alterations associated with HER2+ breast cancer risk and clinical outcome in response to trastuzumab. Mol Biol Rep.

